# Vascular procedures in patients with left ventricular assist devices: single-center experience

**DOI:** 10.1007/s12055-021-01192-3

**Published:** 2021-05-27

**Authors:** Saad Rustum, Julia Neuser, Jan Dieter Schmitto, Thomas Aper, Jasmin Sarah Hanke, Axel Haverich, Mathias Wilhelmi

**Affiliations:** Clinic for Cardiothoracic, Transplantation and Vascular Surgery, Hannover Medical School, Carl-Neuberg-Straße 1, 30625 Hannover, Germany

**Keywords:** Vascular surgery, LVAD, Bleeding, Thrombosis

## Abstract

**Objective:**

A growing number of patients suffering from heart failure is living with a left ventricular assist device (LVAD) and is in the need for non-cardiac surgery. Vascular procedures due to ischemia, bleeding, or other device-related complications may be required and pose a challenge to the caregivers in terms of monitoring and management of these patients. Therefore, we reviewed our experience with LVAD patients undergoing vascular surgery.

**Methods:**

From January 2010 until March 2017, a total of 54 vascular procedures were performed on 41 LVAD patients at our institution. Patient records were reviewed retrospectively in terms of incidence of LVAD-related complications, including thrombosis, stroke, bleeding, wound healing, and survival associated with vascular surgery. The type of surgery was recorded, as well as various clinical demographic variables.

**Results:**

Vascular procedures were performed in 35 men (85.4%) and 6 women (14.6%) with LVADs. There were no perioperative strokes, device thromboses, or device malfunctions. Thirty-day mortality overall was 26.8% (eleven patients), with most patients dying within 30 days after LVAD implantation due to multi-organ failure. In 25 procedures (46.3%), a blood transfusion was necessary.

**Conclusion:**

Patients on LVAD support are a complex cohort with a high risk for perioperative complications. In a setting where device function and anticoagulation are monitored closely, vascular surgery in these patients is feasible with an acceptable perioperative risk.

## Introduction

Left ventricular assist devices (LVADs) have become a viable therapeutic strategy for bridge to transplant (BTT) and destination therapy (DT) in the failing heart [1–4]. With increase in LVAD use and technical progress [5, 6], more patients require non-cardiac surgery or develop complications that are leading to surgical interventions [7–12]. These complications are often related to bleeding, infection, or ischemia and may require a vascular surgical procedure [13, 14]. These patients present multiple challenges to the caregivers and are on long-term anticoagulation with Coumadin and anti-platelet therapy, additionally [15]. Consequently, at the time of surgery, anticoagulation management must balance the potential for thromboembolisms and device thrombosis on the one hand with the risk of bleeding on the other [16, 17]. Previous studies have reported on general non-cardiac surgery in LVAD patients but vascular surgical procedures have not been extensively characterized [10, 18, 19]. Therefore, we reviewed our institutional experience with vascular procedures on patients while on LVAD support.

## Methods

From January 2010 until March 2017, a total of 54 vascular procedures were performed on 41 LVAD patients. During the same time, a total of 498 ventricular assist devices were implanted at our institution and approximately 4800 vascular surgeries were performed. Only 77 (15.5%) patients were women. We reviewed our clinical records retrospectively with a specific focus on incidence of LVAD-related complications, including thrombosis, stroke, bleeding, wound healing, and survival associated with vascular procedures. The study was conducted in accordance with the Declaration of Helsinki. The authors received no specific funding for this work. The type of vascular procedures was recorded as well as the duration of LVAD support at the time of the procedure. Various clinical demographic variables were recorded, including age, sex, etiology of heart failure, peripheral arterial disease (PAD), smoking status, diabetes, renal insufficiency, and the anticoagulation regimen at the time of surgery.

## Data analysis

Data are presented as frequency distributions and percentages. Continuous variables are summarized as mean ± standard deviation or median (range) and were tested for normal distribution with the Kolmogorov-Smirnov test. Differences were analyzed using a *t*-test. If normal distribution was not applicable, the Mann-Whitney *U*-test was performed. Categorical variables were presented in absolute numbers and percentages. For all analysis, a value of *p* < 0.05 was considered statistically significant.

## Results

### Demographics

Vascular procedures were performed in 35 men (85.4%) and 6 women (14.6%) with LVADs. The different assist devices were HeartWare (HeartWare®, Medtronic, MN, USA) in 30 patients (55.6%), HeartMate II (Abbott, Inc, IL, USA) in ten patients (18.5%), and HeartMate III (Abbott, Inc, IL, USA) in one patient (1.9%). Median age was 56 (48–59) years. The etiology of heart failure was non-ischemic dilated cardiomyopathy in 18 patients (43.9%) and ischemic cardiomyopathy in 23 patients (56.1%). Ten patients (24.4%) were suffering from diabetes mellitus and PAD was present in ten patients (24.4%). Twenty-four (58.5%) patients had an active smoking status. A preoperative renal insufficiency was known in 21 patients (51.2%) and ten patients (24.4%) required hemodialysis.

### Types of procedures

There were 54 procedures performed in 41 different patients. A detailed summary of the procedures is presented in Table 1. Thirty-one (57.4%) procedures were for arterial reconstruction, including thrombendarteriectomy and embolectomy, and are presented in Table 2. Procedures related to hemodialysis shunts were done in six patients (11.1%). Amputations (three minor, two major) were necessary in five cases (9.3%) due to vascular complications. In two patients (3.7%), endovascular stenting was performed and ten patients (18.5%) received a catheter-based procedure (tunneled dialysis catheter or distal perfusion catheter while on extracorporeal membrane oxygenation (ECMO) support).

**Table 1:** Summary of vascular procedures

Surgical status	Indication	*n*
Emergency		
Reconstruction of carotid artery and jugular vein	Failed positioning of central venous catheter	1
Thrombendarteriectomy femoral artery +/− PTA or bypass	Acute ischemia of the lower limb	8
Embolectomy (via femoral access)	Acute ischemia of the lower limb	5
Open surgical ECMO implantation	Additional extracorporal support and calcified vessels	2
Shunt resection	High-volume shunt in acute heart failure and bleeding	1
Open surgical distal perfusion catheter placement (on ECMO)	Acute ischemia of the lower limb	3
Explantation of a tunneled catheter	Sepsis	1
Forefoot amputation	Sepsis	1
Elective		
Embolectomy (via femoral access)	Ischemia of the lower limb	4
Thrombendarteriectomy carotid artery	Cartotid artery stenosis	2
Femoral AV-fistula resection	Heart failure	3
Thrombendarteriectomy femoral artery +/− PTA or bypass	Peripheral artery disease	3
Open surgical ECMO explantation	ECMO weaning	3
Stent implantation in LVAD outflowgraft	Suture aneurysm outflowgraft	1
Carotid artery filter implantation	Cerebral protection during LVAD exchange (ventricular thrombus)	1
Shunt (implantation or revision)	Hemodialysis	5
Tunneled dialysis catheter implantation	Hemodialysis	6
Toe amputation	Necrosis	2
Leg amputation	Peripheral artery disease (Fontaine IV)	2

**Table 2:** Reconstructive vascular surgery

*n*	31
Emergency	16
Age	56.5 (48.2–59.5)
BMI	25.2 (23.1–26.8)
Sex (male)	23 (74.2%)
Diabetes	7 (22.5%)
PAD	7 (22.5%)
Etiology of heart failure DCM	10 (32.2%)
Renal insufficiency Dialysis	12 (38.7%) 3 (9.7%)
Smoker	16 (51.6%)
INR pre	1.79 ± 0.6
Pre-operative ASS	6 (19.4%)
Pre-operative clopidogrel	9 (29.0%)
Within 3 days of LVAD implantation Duration of support	9 (29.0%) 193 (3–772)
On ECMO support	7 (22.5%)
General anesthesia	31 (100.0%)
Duration of surgery	93.5 (50–131)
Perioperative transfusion	9 (29.0%)
Surgical re-exploration	0 (0%)
Stroke	0 (0%)
Device malfunction	0 (0%)
Wound complications	5 (16.1%)
30-d mortality in elective procedures	0 (0%)

Abbreviation: *BMI*, body mass index

### Timing of surgery

Twenty-two cases (40.7%) were performed as an emergency procedure and eleven surgeries (20.4%) have been performed within 3 days after LVAD implantation. In 14 procedures (25.9%), the patient was on ECMO support in addition to the assist device. During 44 (81.5%) surgeries, an arterial line was placed to monitor the blood pressure; in ten cases (18.5%), a cuff sufficed when the patients had appropriate pulsatility.

### Morbidity and mortality after vascular surgery

There were no perioperative strokes, device thromboses, device malfunctions, or surgical re-explorations due to bleeding. Thirty-day mortality overall was 26.8% (eleven patients), with most patients dying within 30 days after LVAD implantation due to multi-organ failure. In 25 procedures (46.3%), a blood transfusion (packed red blood cells, PRBCs) was necessary. Surgical re-exploration due to bleeding did not occur; however, in nine patients (16.7%), there was prolonged wound healing.

### Comparison of elective and emergency procedures

The results of this comparison are presented in Table 3. There was a significant difference in preoperative international normalized ratio (INR) (*p* = 0.021) with a higher INR before emergency procedures. A significant number of emergency procedures was performed within 3 days of LVAD implantation (*p* = 0.017) as well as on ECMO support (*p* = 0.039). The necessity for transfusion of PRBCs was higher in emergency procedures (68.2% vs. 31.3%). The 30-day mortality was also higher in patients requiring emergent surgery (40.9% vs. 6.3%). Comorbidities, duration of surgery, and complexity of procedures are comparable between the groups.

**Table 3:** Comparison of emergency and elective procedures

	Emergency	Elective	*p*-value
*n*	22	32	
Age	56.5 (51.8–61)	56.0 (50–59)	0.744
BMI	26.2 ± 4.4	24.4 (23.2–30.3)	0.951
Sex (male)	20 (90.9%)	29 (90.6%)	0.972
Diabetes	6 (27.3%)	9 (28.1%)	0.946
PAD	4 (18.2%)	12 (37.5%)	0.130
Etiology of heart failure			
DCM	11 (50%)	14 (43.8%)	0.654
ICM	11 (50%)	18 (56.3%)	0.373
Renal insufficiency Dialysis	10 (45.5%) 4 (18.2%)	22 (68.7%) 13 (40.6%)	0.090 0.084
Smoker	12 (54.5%)	15 (46.9%)	0.583
INR pre	1.99 ± 0.7	1.46 ± 0.3	0.021
Pre-operative ASS	3 (13.6%)	5 (15.6%)	0.841
Pre-operative clopidogrel	8 (36.4%)	14 (43.8%)	0.591
Within 3 days of LVAD implantation	8 (36.4%)	3 (9.4%)	0.017
Duration of support	236 (2–886)	337 (53–643)	0.413
On ECMO support	9 (40.9%)	5 (15.6%)	0.039
General anesthesia	21 (95.5%)	27 (84.4%)	0.207
Duration of surgery	57 (38–113)	93.3 ± 55	0.202
Type of surgery			
Vascular reconstruction Stent Shunt Catheter-based procedure Amputation	16 (72.7%) 0 (0%) 1 (4.5%) 4 (18.2%) 1 (4.5%)	15 (46.9%) 2 (6.3%) 5 (15.6%) 6 (18.8%) 4 (12.5%)	0.061 0.236 0.207 0.958 0.326
Perioperative transfusion	15 (68.2%)	10 (31.3%)	0.008
Surgical re-exploration	0 (0%)	0 (0%)	
Stroke	0 (0%)	0 (0%)	
Device malfunction	0 (0%)	0 (0%)	
Wound complications	6 (27.3%)	3 (9.4%)	0.348
30-d mortality	8 (36.4%)	2 (6.3%)	0.002

### Comparison of patients who did and did not require transfusion of PRBCs

Results of the analysis regarding the necessity of PRBC transfusion are presented in Table 4. A significant number of procedures where PRBCs were administered was performed within 3 days of LVAD implantation (*p* = 0.009), on ECMO support (*p* ≤ 0.001), or as emergency cases (*p* = 0.008). All patients who received blood have been under general anesthesia and more complex vascular reconstructions (80.0%) have been performed. INR was comparable in both groups (1.57 vs. 1.48); however, a higher number of patients who did not receive a PRBC transfusion was on anti-platelet therapy with clopidogrel (*p* = 0.021).

**Table 4:** Comparison of patients who did and did not require PRBC transfusion

	Required PRBCs	No PRBCs required	*p*-value
*n*	25	29	
Age	56 (52–60)	56 (51–60)	0.869
BMI	25.3 ± 4.68	26.3 (23.5–30.7)	0.150
Sex (male)	23 (92.0%)	26 (89.7%)	0.769
Diabetes	7 (28.0%)	8 (27.6%)	0.973
PAD	8 (32.0%)	8 (27.6%)	0.726
Etiology of heart failure			
DCM ICM	15 (60.0%) 10 (40.0%)	10 (45.5%) 19 (65.6%)	0.063 0.031
Renal insufficiency Dialysis	13 (52.0%) 4 (16.0%)	19 (65.5%) 13 (44.8%)	0.381 0.024
Smoker	14 (56.0%)	13 (44.8%)	0.417
INR	1.48 (1.23–1.93)	1.57 (1.38–1.78)	0.768
Pre-operative ASS	2 (8.0%)	6 (20.7%)	0.198
Pre-operative clopidogrel	6 (24.0%)	16 (55.2%)	0.021
Within 3 days of LVAD implantation	9 (36.0%)	2 (6.9%)	0.009
Duration of support	63 (49–115)	373 (201–768)	0.077
On ECMO support	12 (48.0%)	2 (6.9%)	<0.001
Emergency	15 (60.0%)	7 (24.1%)	0.008
General anesthesia	25 (100%)	23 (79.3%)	0.017
Duration of surgery	95.4 ± 54	56 (14–169)	0.077
Type of surgery			
Vascular reconstruction Stent Shunt Catheter-based procedure Amputation	20 (80.0%) 1 (4.0%) 1 (4.0%) 2 (8.0%) 1 (4.0%)	11 (37.9%) 1 (3.4%) 5 (17.2%) 8 (27.6%) 4 (13.8%)	0.002 0.916 0.126 0.067 0.220
Surgical re-exploration	0 (0%)	0 (0%)	
Stroke	0 (0%)	0 (0%)	
Device malfunction	0 (0%)	0 (0%)	
Wound complications	5 (20.0%)	4 (13.8%)	0.916
30-d mortality	8 (32.0%)	2 (6.9%)	0.051

## Discussion

With a growing number of implanted LVADs, especially as a destination therapy, patients are older and present with more comorbidities [7, 20, 21]. On the other hand, younger patients on bridge to transplant therapy live a more active lifestyle and require vascular surgery due to peripheral artery disease which might limit their everyday activities. Therefore, an increasing number of patients is in the need for non-cardiac surgical procedures. In this study, we present the outcomes of vascular interventions in patients on a left ventricular assist device who have been operated at our institution. To our notice, our cohort of 41 patients, where 54 vascular procedures were performed, is one of the largest cohorts examined [18, 19, 22].

Several aspects of LVAD therapy must be considered when performing vascular surgeries in these patients, including high levels of anticoagulation or acquired von Willebrand disease [11, 23]. The mean INR of our entire cohort was 1.68 ± 0.58 and therefore in the therapeutic range where an extremely low frequency of thromboembolic events has been reported [24]. In addition, anti-platelet therapy either with aspirin (8 patients) or clopidogrel (22 patients) was administered. When comparing elective and emergency vascular procedures in LVAD patients at our institution, the INR was significantly higher in an emergency setting, while anti-platelet therapy was comparable. There was a significantly higher rate of PRBC transfusion in the emergency group (68.2% vs. 31.3%). However, when comparing patients who received and who did not receive PRBCs perioperatively, no significant difference was found in the INR at the time of surgery. When analyzing the patients who underwent an emergency vascular procedure, a substantial number was on simultaneous ECMO support (40.9%), and in 36.4%, the surgery was performed within the first 3 days after LVAD implantation. We believe that this constellation has led to the significantly higher rate of PRBC transfusion in emergency procedures. It is also an explanation for the significantly higher 30-day mortality rate (36.4% vs. 6.3%) in the emergency group. The patient cohort that did not require a PRBC transfusion had a significant higher rate of patients receiving clopidogrel (*p* = 0.021) as additional anti-platelet therapy. It seems that whether patients were given aspirin or clopidogrel had no substantial influence on perioperative bleeding in our cohort. In addition, the majority of procedures (80%) in the group that received a blood transfusion were complex vascular reconstruction (*p* = 0.002).

The types of procedures performed in an elective setting were analogous to the procedures performed urgently. Surgical re-exploration due to bleeding was not required in any patient; at the same time, none of our patients suffered from stroke or device thrombosis with resulting device malfunction. However, there was a 30-day mortality of 18.5% overall. Considering that 39 of our vascular procedures have been performed within the first month of LVAD implantation, and a total of 14 procedures were performed on simultaneous ECMO support, the mortality rate is for the most part due to complications regarding the heart failure and is within the reported mortality range after LVAD implantation [25–27].

There was prolonged wound healing in a total of nine cases (4.9%) which entailed escalated or extended antibiotic therapy. Moreover, those patients were seen by our wound managers (registered nurses with special training in wound care) on a daily basis. Wound complications were mostly observed after femoral access, especially in patients with a higher body mass index (BMI). In this high-risk patient cohort, we anticipated a prolonged wound healing; therefore, we used special wound dressing prophylactically (e.g., antibacterial dressing). Although surgical re-exploration was not necessary, meticulous wound care in LVAD patients is vital to avoid blood stream infections and further device complications [28].

### Limitations

A limitation of this study is the heterogeneity of the performed vascular procedures with different risks and complication rates. The study has been performed retrospectively, was non-randomized, and only reflects a single-center experience.

## Conclusion

Our study represents the largest number of cases where vascular intervention is performed in patients on LVAD support. It underlines that vascular surgery after LVAD is feasible as long as ventricular assist device (VAD)-specific pitfalls are addressed. Besides the heart failure with all its consequences, the anticoagulation regimen and acquired von Willebrand disease can cause further complications. In an experienced center where device function is monitored closely perioperatively, vascular surgery in LVAD patients can be performed safely with a low rate of complications.
